# Targeting the endocannabinoid system to treat haunting traumatic memories

**DOI:** 10.3389/fnbeh.2013.00124

**Published:** 2013-09-19

**Authors:** Irit Akirav

**Affiliations:** Department of Psychology, University of Haifa Haifa, Israel

**Keywords:** endocannabinoids, memory, PTSD rat, anxiety disorders, extinction learning

One of the core symptoms in post-traumatic stress disorder (PTSD) is the traumatic memory that constantly haunts the patient. PTSD is a paradoxical disorder of memory—on the one hand, intrusive recollections for certain aspects of the event and, on the other hand, amnesia for other important aspects (Ehlers et al., 2004). Intrusive memories, often triggered automatically by situations that reflect aspects of the traumatic event, are accompanied by high levels of arousal and might be experienced as “flashbacks” (vanOyen Witvliet, 1997).

A major question in studying PTSD is how can an external traumatic event impact on the biology of the organism to produce such durable memory traces of that event that are so highly resistant to extinction? (Pitman, 1989).

An increasing body of evidence points to the endocannabinoid (eCB) system as a key system in the regulation of emotionality and memory (Haller et al., 2002, 2004; Marsicano et al., 2002; Lutz, 2007; Ganon-Elazar and Akirav, 2009; Abush and Akirav, 2010; Moreira and Wotjak, 2010; Parolaro et al., 2010; Atsak et al., 2012b; Ganon-Elazar and Akirav, 2012, 2013; Abush and Akirav, 2013; Campolongo et al., 2013). Hence, eCB enhancers may be the ideal pharmacological treatment for PTSD by blocking the pathological over consolidation and continuous retrieval of the traumatic event on the one hand, and enhancing its extinction and reducing the anxiety symptoms on the other hand. These effects fit well with the concept of reducing fear memory.

These ideas were recently published in an interesting and highly relevant perspective by Trezza and Campolongo. The authors suggested the eCB system as a possible pharmacological treatment that modulates the consolidation, retrieval and extinction of these durable memory traces. Together with the effects of eCB on stress and anxiety, the whole PTSD spectrum is addressed.

The authors reviewed the literature suggesting that eCBs have an essential role in maintaining emotional homeostasis (Haller et al., 2002, 2004; Lutz, 2007; Niyuhire et al., 2007; Hill and Gorzalka, 2009; Moreira and Wotjak, 2010; Parolaro et al., 2010) and in modulating memory consolidation, retrieval and extinction (Marsicano et al., 2002; Niyuhire et al., 2007; Marsicano and Lafenetre, 2009; Atsak et al., 2012a; Campolongo et al., 2013). Specifically, cannabinoids strongly facilitate memory extinction in animals (Marsicano et al., 2002; Lutz, 2007; Abush and Akirav, 2010), while impairing memory retrieval (Niyuhire et al., 2007; Atsak et al., 2012a). Regarding the effects of cannabinoids on memory consolidation for emotionally salient events, they reported controversial results from preclinical studies. On the one hand, post-training administration of cannabinoid receptor direct or indirect agonists facilitated memory consolidation in the inhibitory avoidance task (Campolongo et al., 2009; Hauer et al., 2011). However, other studies suggested that cannabinoid agonists administered to rats shortly after exposure to a series of intense stressful events prevented PTSD-like symptoms (Ganon-Elazar and Akirav, 2012, 2013).

Hence, the authors concluded that eCBs could be an ideal drug to treat PTSD by addressing both the emotional and cognitive aspects of the disorder. Yet, attention needs to be dedicated to the time framing of pharmacological treatment, with an attempt to avoid the first early phases of memory consolidation.

Indeed, accumulating data from both clinical and pre-clinical studies suggest that targeting the eCB system may benefit PTSD. Given the similarities between extinction procedures and exposure-based psychotherapy used for the treatment of fear disorders in humans (Myers and Davis, 2007), the eCB system represents a novel pharmacological target for anxiety disorders related to inappropriate retention of aversive memories (Marsicano et al., 2002; Chhatwal et al., 2005; Bitencourt et al., 2008). Accordingly, human studies suggest that cannabinoids may be used as an adjunct to extinction-based therapies for anxiety disorders. In support, Rabinak et al. (2013) found that subjects that received Δ ^9^–THC showed enhanced extinction memory and Das et al. (2013) showed a similar effect using the agonist cannabidiol.

A recent study with rats suggested that predator exposure causes long-lasting anxiogenic effects associated with hyperactivation of amygdaloid complex and modulation of CB1 receptor in brain areas related to PTSD symptoms (Campos et al., 2013). Seven days after predator threat rats showed lasting anxiogenic effects and CB1 mRNA expression was down regulated in the frontal cortex and amygdaloid complex (Campos et al., 2013). Studies in humans also suggested alterations in the eCB system in PTSD. Hauer et al. (2013) indicated that individuals with PTSD show significant differences in plasma concentrations of eCBs and related N-acyl-ethanolamides when compared to healthy controls and to subjects after trauma exposure who did not develop PTSD. Neumeister et al. (2013) reported that PTSD patients demonstrated elevated brain cannabinoid CB1 receptor availability and suggested that abnormal CB1 receptor-mediated anandamide signaling is implicated in the etiology of PTSD. Moreover, it has been suggested that higher CB1 receptor density in women than men under basal (i.e., nonstress) conditions may explain the epidemiological literature consistently reporting gender differences in PTSD. Hence, higher CB1 receptor density in women may be a risk factor to developing PTSD after trauma (Neumeister, 2013).

Cannabinoid agonists administered shortly after exposure to an intense stressful event (i.e., exposure to 2 h restraint followed by forced swim and sedation or exposure to predator stress) can prevent the development of PTSD-like symptoms in rats (Campos et al., 2012; Ganon-Elazar and Akirav, 2012, 2013), suggesting that eCB enhancers may be beneficial in PTSD. Indeed, two open label clinical trials demonstrated potential benefits of cannabis in patients with PTSD. Fraser (2009) found that the synthetic cannabinoid nabilone significantly improved treatment-resistant nightmares in PTSD patients. Shalev's (Shalev et al., 2013) group reported that 10 outpatients with chronic PTSD that received Δ ^9^–THC twice a day for 3 weeks demonstrated a significant improvement in arousal, sleep quality and nightmares.

Several studies support the self-medication hypothesis explanation for cannabis use to cope with PTSD symptoms (Cougle et al., 2011; Passie et al., 2012). Others reported a strong correlation between PTSD symptom severity and the amount of cannabis use (Bonn-Miller et al., 2011; Cougle et al., 2011; Potter et al., 2011). Moreover, the starting point of using cannabis correlated with the onset of PTSD symptoms (Cougle et al., 2011) suggesting that cannabis use was used to help reduce aversive mood states.

To conclude, the eCB system may be a useful target for treating both the cognitive and emotional features of PTSD, but more research is needed in order to recognize and avoid the circumstances under which a pharmacological treatment with eCB agents might have a deleterious effect on behavior.
